# Catalytic Design of Matrix-Isolated Ni/Chitosan Composites for Methane Decomposition

**DOI:** 10.3390/ijms27031255

**Published:** 2026-01-27

**Authors:** Anastasiia Sotnikova, Mikhail Ivantsov, Valeriia Vasileva, Mayya Kulikova

**Affiliations:** Topchiev Institute of Petrochemical Synthesis, Russian Academy of Sciences, Leninsky Prospekt, Bld. 29, 119991 Moscow, Russia; ivantsov@ips.ac.ru (M.I.); vasileva_v_v@ips.ac.ru (V.V.); m_kulikova@ips.ac.ru (M.K.)

**Keywords:** catalytic design, composite, polymer matrix, chitosan, Ni/C catalysts, methane catalytic decomposition

## Abstract

Targeted synthesis of Ni/C-containing composite materials was carried out using the matrix isolation method. The Ni content was varied (5–20 wt.% from chitosan). The morphology and physicochemical properties of the obtained materials were characterized using a number of methods: elemental analysis, SEM, TEM, XRD, FTIR, Raman spectroscopy, TPR–H_2_, and SSA. FTIR showed that nickel ions are immobilized on the chitosan molecule, and heat treatment of the polymer molecule results in the formation of polyconjugation centers. It was also revealed that heat treatment of the salt–polymer films results in the formation of a graphite-like structure (Raman spectroscopy) with the inclusion of nickel in metallic form (XRD, TPR–H_2_), with a particle size from 2 to 10 nm (TEM). The composites were shown to have a SSA of up to 269 m^2^/g. The resulting composite materials were used as catalysts for the decomposition of methane to produce hydrogen. High activity was observed in the catalytic methane decomposition at 700 °C (methane conversion up to 25.8%; hydrogen yield up to 1.98 g_H2_/g_Ni_/h).

## 1. Introduction

The field of nanotechnology and nanomaterial synthesis is currently actively developing, making it possible to take into account and balance certain economic and social aspects. The introduction of nanotechnology has helped improve such fundamental areas of industry as electronics and information systems, medicine, and biological sciences, as well as energy and environmental safety [1]. This field is a relevant topic at the present time [2,3,4]. In this regard, the synthesis of nanoscale materials is a key priority in the development of modern science. An analysis of current trends in the field of nanomaterials reveals two of the most pressing challenges faced by scientists: the development of a methodology for obtaining new nanomaterials and a comprehensive study of their physicochemical properties, followed by the determination of their areas of application [5,6,7].

One of the key areas where the solution and improvement of these problems could lead to significant progress is heterogeneous catalysis. Materials for heterogeneous catalysis, despite many advantages, namely, mechanical strength, chemical stability, and ease of preparation, are inferior to homogeneous materials in the main process parameters, for example, in selectivity and conversion. The presence of these disadvantages necessitates the development of new heterogeneous materials [8,9,10,11]. An important criterion for the creation of new materials is the selection of the most effective support. Metal oxides, zeolites, and carbon supports are mainly used as a base [12,13]. The choice in favor of these systems is determined by their high chemical and thermal stability.

Recently, research using polymers with immobilized metal nanoparticles as carriers has been actively developing [14,15,16,17]. For example, in the work of Wissing M. et al. [18], gold and palladium nanoparticles on zeolite, combined with photoactive polymer brushes, were developed. In the work of Nagarjuna R. et al. [19], a Pd-containing catalyst was synthesized using polyethyleneimine based on Al_2_O_3_ with subsequent adsorption of palladium.

Scientific interest is currently shifting towards the search for and development of environmentally friendly and renewable alternatives that will be able to replace classical synthetic polymers. One such area of modern research, which meets the principles of green chemistry, is the creation of safe nanomaterials from renewable sources [20]. Taking into account all the requirements for safe materials, natural polysaccharides are promising in this area. Due to the diversity of reactive functional groups in their structure, polysaccharides are capable of forming strong composite materials with various inorganic materials and metal ions [21,22,23]. Thus, in the work of de Almeida D.A. et al. [24], scientists synthesized hydrogel composites based on pectin with gold nanoparticles. In the work [25], palladium nanoparticles stabilized with gelatin/pectin were obtained as materials.

Among the wide variety of polysaccharides, chitosan demonstrates the greatest potential in the creation of nanocomposite materials. Chitosan, as a natural polysaccharide, attracts attention due to the combination of several properties: high chemical activity, non-toxicity, renewableness, and availability [26]. Chitosan is studied as a complexing and stabilizing agent in the synthesis of catalytic systems [27]. Due to the presence of active amino groups, thermal stability, and inertness in organic media, chitosan is a multifunctional matrix for the immobilization of metal-containing nanoparticles intended for use in catalysis. The incorporation of metal nanoparticles into the polymer matrices of composite materials can improve the properties of these materials, namely, increase chemical and thermal resistance and provide higher mechanical properties [28,29]. Metal/polymer composites can be obtained by matrix isolation, where the polymer acts as a stabilizer for metal nanoparticles. This method allows for preventing the coagulation of metal nanoparticles while maintaining their properties [30].

When selecting an active metal for immobilization in a polymer matrix, transition metals are of particular interest. Group VIII metals are often used in gas chemistry processes due to the presence of partially filled 3 d-electron orbitals that are capable of accepting additional electrons. Thus, in [31], a series of nano-catalysts with the composition Fe5–Co–Zn/Al_2_O_3_ with varying stoichiometric coefficients of Co and Zn were synthesized for the CH_4_ decomposition process. In another study [32], bimetallic MoNi nanoparticles supported on MgO were also investigated in the methane decomposition process. The use of nickel as an active metal is the most promising since it is more reactive than Fe and Co and operates at lower temperatures in the methane decomposition process [33,34]. In addition to the metals listed above, other elements capable of immobilization on carbon supports can also serve as active components. For example, Ref. [35] described a photothermal catalyst based on a stable and uniformly dispersed graphene-like biomaterial, in the matrix of which atomically dispersed neighboring potassium atoms are immobilized. The resulting system has been successfully used for the catalytic production of biodiesel fuel. Another study [36] presented a highly efficient Bi_1−x_VO_4_—Cu catalytic system, where copper atoms are fixed to bismuth vacancies in the BiVO_4_ structure. This catalyst demonstrates exceptional activity in the electrocatalytic synthesis of methane from CO_2_ with a high kinetic isotope effect and high specific productivity.

Today, there are many environmental problems in the world, one of which is the emission of greenhouse gases [37,38]. To reduce environmentally harmful CO_x_ emissions, a method for catalytic decomposition of methane (CDM) was developed, which allows for obtaining “turquoise” hydrogen and carbon in solid form. The classic temperature of methane decomposition is 1200 °C, while in CDM, the temperature is 700–800 °C [39,40,41]. Catalysts for the catalytic decomposition of methane are divided into two main groups: metal-containing and carbon materials. One of their main differences is the temperature regime: carbon materials are active from 800 °C, while metal-containing systems are active in a lower range of 600–700 °C [42,43]. Carbon materials, in turn, have high stability, which is due to the developed specific surface area. Metal-containing catalysts, despite high activity, are subject to deactivation due to carbonization, which negatively affects the process [44,45]. To prevent these disadvantages, attempts are being made to combine metal-containing and carbon materials that will combine a developed specific surface area and high activity.

In this study, composite materials based on chitosan with added nickel were synthesized for the first time using matrix isolation in an inert atmosphere and characterized. The catalytic activity of the resulting Ni-containing carbon composites was studied in the methane decomposition reaction.

## 2. Results and Discussion

### 2.1. Characteristics of Materials

#### 2.1.1. Elemental Analysis

Table 1 presents a list of the resulting composites and their elemental composition. The nickel content was varied across the catalyst line. To achieve this, the amount of nickel nitrate was increased, and the Ni/chitosan ratio was varied as follows: 1/20 → 2/20 → 3/20 → 4/20, corresponding to Ni contents of Ni 5, 10, 15, and 20 wt.% from chitosan. After heat treatment of the catalyst precursors, an increase in the Ni content relative to the initial one (~2 times) is observed. The highest nickel content is characteristic of the Ni/Ch–4 sample, which reaches 47.5 wt.%. Also, with an increase in the nickel content in the composites, a decrease in the content of carbon (from 61.7 to 40.7 wt.%), nitrogen (from 11.3 to 6.7 wt.%), and hydrogen (from 2.1 to 1.1 wt.%) is observed.

#### 2.1.2. X-Ray

**Figure 1:**
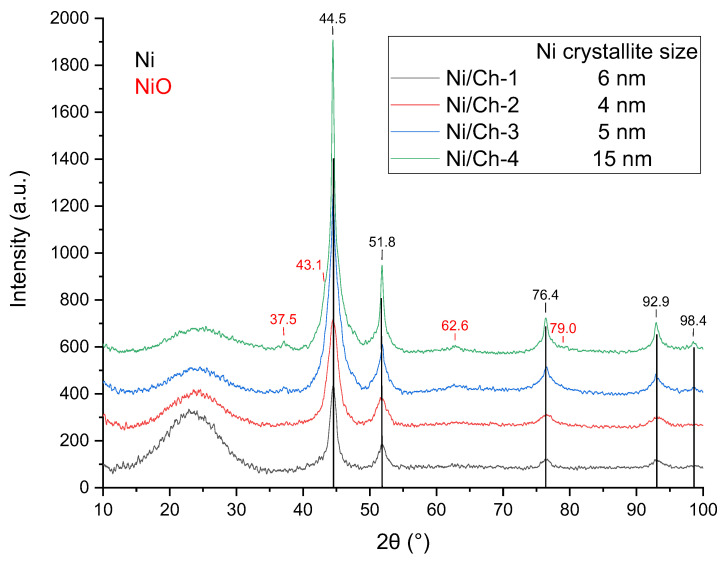
XRD of composite materials.

The X-ray diffraction patterns of the composite materials are shown in Figure 1. During heat treatment, all samples were characterized by the formation of the Ni phase (PDF#65–2865, 2θ = 44.5°, 51.8°, 76.4°, 92.9°, 98.4°). Also, for the Ni/Ch–3 and Ni/Ch–4 samples, the appearance of the NiO phase can be judged by the following low-intensity peaks 2θ = 37.5°; 41.8°; 62.8°; 79.0° (PDF#89–7130). The stability of the Ni phase formed during the catalyst synthesis is possibly due to the fact that most of the metal is covered with carbon. And the presence of the NiO phase in the Ni/Ch–3 and Ni/Ch–4 samples is probably due to the insufficient amount of polymer destruction products for the complete reduction of NiO to Ni. The low intensity of the NiO phase reflections is likely due to either the X-ray amorphous nature of this phase or the excessively small particle size. The X-ray diffraction results also suggest the formation of a highly dispersed system. The average nickel crystallite size was 4–6 nm for all catalyst samples except Ni/Ch–4, for which the Ni crystallite size was 15 nm, likely due to the high thermal mobility of the metal.

#### 2.1.3. TPR–H_2_

**Figure 2:**
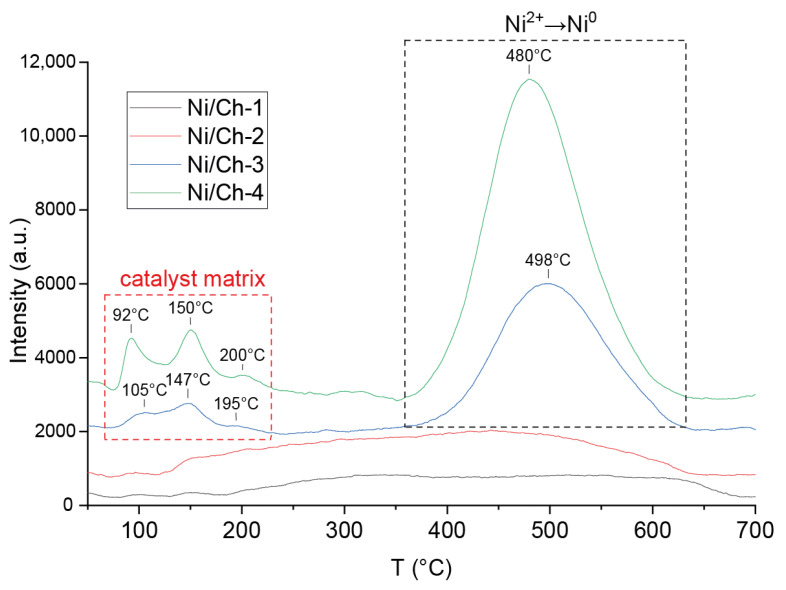
TPR–H_2_ profile of samples of composite materials.

The TPR–H_2_ spectra for the catalysts are shown in Figure 2. According to the TPR–H_2_ data for the Ni/Ch–3 and Ni/Ch–4 composite material samples, two main reduction peaks are observed for the Ni/Ch–3 and Ni/Ch–4 composite material samples, one in the temperature range of 75–200 °C and the other with a maximum at about 500 °C. The first reduction peak is due to hydrogen absorption by the carbon matrix, which results in the removal of oxygen and nitrogen atoms from the catalyst. The peaks in the 500 °C region correspond to the temperature range of NiO reduction to metallic nickel (Ni^2+^ → Ni^0^). Moreover, the amount of absorbed hydrogen for the Ni/Ch–4 sample is 1.8 times greater than for Ni/Ch–3. However, for the Ni/Ch–1 and Ni/Ch–2 catalyst samples, virtually no hydrogen absorption is observed in the 500 °C region, which is likely due to the fairly complete reduction of NiO during the catalyst synthesis. It can be concluded that the released H_2_ and CO, when exposed to thermal stress on the metal–polymer film, reduce NiO to metallic nickel (Ni^2+^ → Ni^0^), but with an increase in the NiO content in the precursor, H_2_ and CO are not enough for reduction, and some of the NiO remains after thermal stress, which is consistent with the XRD results (Figure 1).

#### 2.1.4. IR Spectroscopy

Figure S1 (Supplementary Materials) shows the IR spectra of the initial chitosan and the salt–polymer films, and Table 2 presents the wavenumbers of the main bands. The spectrum of chitosan is similar to the spectra presented in [46,47,48]. In the IR spectrum of chitosan, a broad band with a maximum at 3366 cm^−1^ corresponds to the stretching vibrations of –NH_2_ and –OH. For the salt–polymer films, a shift in this band to the region of lower frequencies (~3310 cm^−1^) is observed, which indicates that the –NH_2_ and –OH groups are involved in complexation [49]. The appearance of a new peak in the region of 3177–3180 cm^−1^, the intensity of which increases with increasing nickel content, can also be associated with the redistribution of hydrogen bonds due to complexation. The wavenumbers of the chitosan bands with maxima at 2914 and 2874 cm^−1^ correspond to the –CH_2_ stretching vibrations in the pyranose ring. With the addition of Ni(NO_3_)_2_, the wavenumbers related to the –CH_2_ stretching vibrations change only slightly. The formation of a chelate complex is also indirectly confirmed by the absorption bands corresponding to the amide bands. Thus, with the introduction of a nickel salt into the polymer, a shift in the C=O (amide I) absorption band to longer wavelengths (from 1653 to 1601 cm^−1^) is observed. A similar shift in the absorption band is characteristic of NH deformation vibrations. The appearance of absorption bands with maxima in the range of 1630–1600 cm^−1^ and 1545–1555 cm^−1^ is considered as a characteristic peak of the association between chitosan and metal [49,50]. Also, in the spectra of salt–polymer films, the appearance of an absorption band with a maximum at 827 cm^−1^ is characteristic. The appearance of this band is associated with the introduction of nickel nitrate and the formation of various nitro compounds [51].

**Figure 3:**
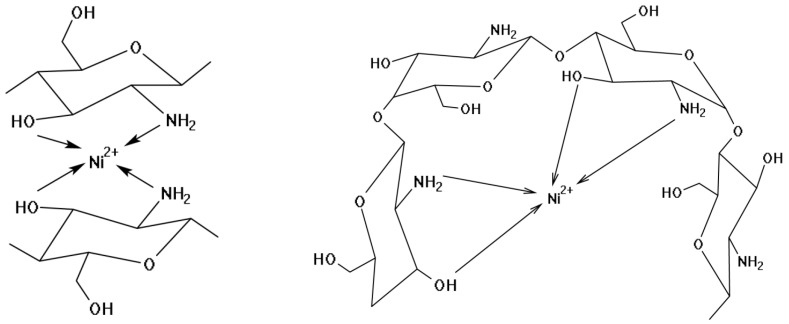
Proposed structures of the fragment formed during the interaction between Ni^2+^ and chitosan.

Based on the analysis of the IR spectra of chitosan and precursors of composite materials, it can be assumed that a chelate complex compound is formed (Figure 3).

**Figure 4:**
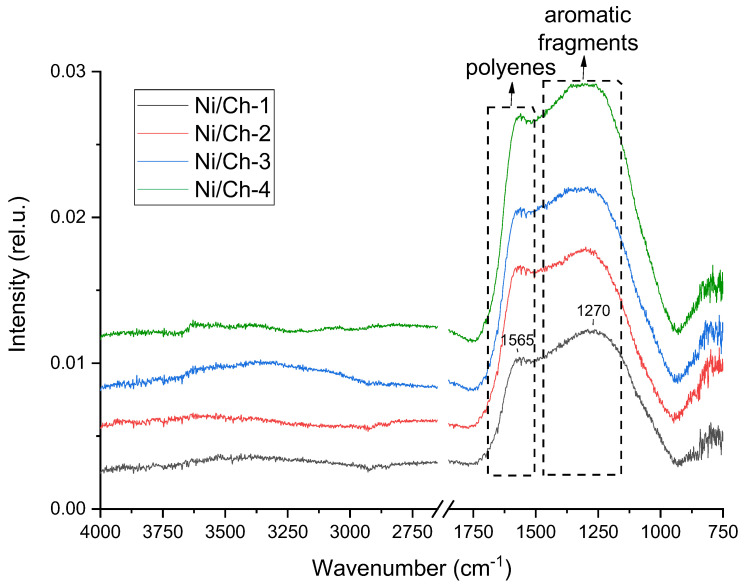
IR Fourier spectroscopy of composite materials obtained by thermal decomposition of a mixture of nickel nitrate and chitosan.

Figure 4 shows the IR spectra of the composite materials after heat treatment in an Ar environment at 500 °C. It was found that heat treatment in an inert environment leads to the disappearance of the absorption bands in the region of OH and NH stretching vibrations (~3300 cm^−1^), as well as in the region of aliphatic structure stretching vibrations (~2900 cm^−1^). The absence of these absorption bands indicates profound destruction of chitosan, including decomposition of aliphatic structures. An intense broad absorption band is observed with a maximum at 1565 cm^−1^ and mutually overlapping bands in the region of 1100–1500 cm^−1^. The 1565 cm^−1^ band is probably due to stretching vibrations of C=C bonds in polyene chains, which are weakly active in the IR range [52]. And the overlapping bands in the region of 1100–1500 cm^−1^ are characteristic of various aromatic fragments [53].

#### 2.1.5. Raman Spectroscopy

**Figure 5:**
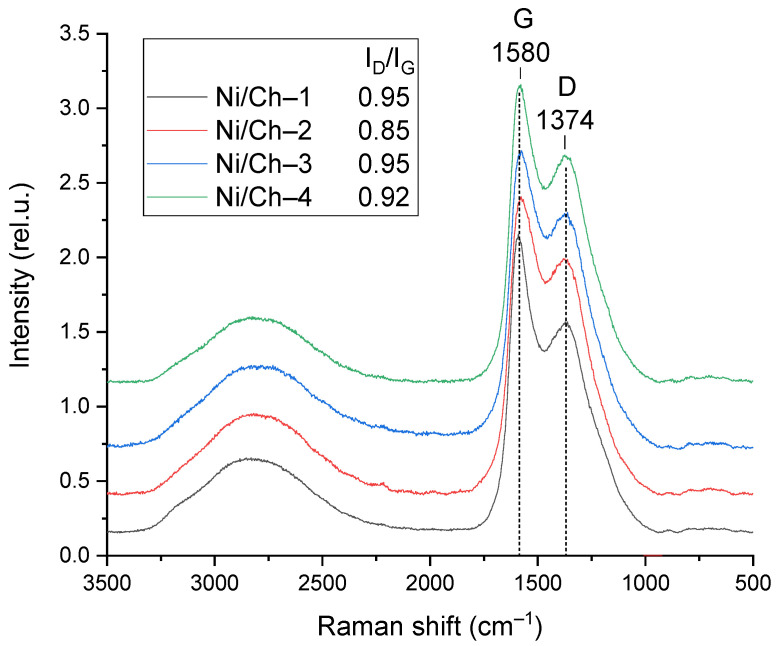
Raman spectra of composite materials.

The composite materials were studied using Raman spectroscopy. The obtained spectra are shown in Figure 5. The Raman spectra contain two main typical bands characteristic of graphite-like materials. The G band has a maximum at ~1580 cm^−1^ and characterizes carbon in sp^2^-hybridization, characteristic of the C=C bond. The spectra also contain a band with a maximum at ~1374 cm^−1^, characteristic of the D band, caused by the presence of defects and corresponding to sp^3^-hybridization. Overlapping bands in the region of 3300–2300 cm^−1^ can be attributed to unresolved overtones of the D and G bands [54]. To analyze the structural defects, the intensity ratio of the D and G bands (I_D_/I_G_) was calculated, which is a key parameter for assessing the degree of defects. I_D_/I_G_ for all studied samples varied within a narrow range from 0.85 to 0.95. The obtained data show that increasing the mass fraction of nickel in the composite does not lead to a significant change in the I_D_/I_G_ value. This indicates that varying the Ni content within the studied limits does not have a significant effect on the defect density in the forming carbon matrix.

#### 2.1.6. Morphology Characterization

The textural characteristics of the composite samples are presented in Table 3. For context, the specific surface area of the pure chitosan support subjected to the same thermal treatment is negligible (0.2 m^2^/g), highlighting the critical role of nickel in the pore-forming process. The obtained data demonstrate a clear dependence of the porosity parameters on the composition. The total specific surface area (S_sp_) increases sharply from 26 m^2^/g for the Ni/Ch–1 sample to 257 m^2^/g for Ni/Ch–2. This indicates a sharp activation of the surface with an increase in the Ni(NO_3_)_2_ content in the composite precursors. A further increase in the Ni content also leads to changes. The surface reaches a maximum of 269 m^2^/g for Ni/Ch–3, and then decreases to 224 m^2^/g for the Ni/Ch–4 sample. A similar nonlinear dependence is observed for the contribution of micropores: their area (S_micro_) and volume (V_micro_) are maximum for the Ni/Ch–2 sample (244 m^2^/g and 0.11 mL/g) and decrease noticeably with a further increase in the nickel content. Moreover, the parameters associated with mesopores (S_meso_, V_meso_) and the outer surface (S_ext_) steadily increase with increasing Ni content. This leads to a consistent increase in the total pore volume (V_pore_), but the average pore diameter (D) also varies nonlinearly, which is related to the distribution of micro- and mesopores.

The observed dynamics are likely related to the activation of the carbon component of the composites and are induced by Ni(NO_3_)_2_ [55]. During heat treatment of the salt–polymer films, the released gaseous nitrogen oxides act as an activating agent. The low Ni content (5 wt.% from chitosan) in the Ni/Ch–1 composite precursor proved insufficient for effective activation. A Ni concentration of 10 wt.% from chitosan (Ni/Ch–2) leads to intense but controlled gas evolution, forming a developed microporous surface with a minimal average pore size. Increasing the nickel content (Ni/Ch–3, Ni/Ch–4) causes more aggressive gas evolution, leading to partial destruction of the walls between micropores, their enlargement, and the formation of mesopores. This process explains the observed shift in pore distribution and a significant increase in average pore diameter. Thus, by varying the nickel content, it is possible to specifically control not only the specific surface area but also the pore size distribution in the composite material.

**Figure 6:**
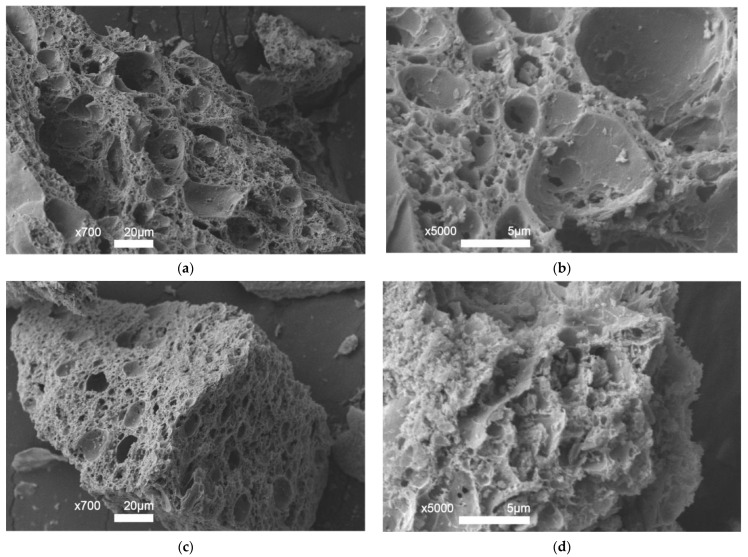
SEM images of composite materials: (**a**,**b**) Ni/Ch–2; (**c**,**d**) Ni/Ch–4.

Figure 6 shows scanning microscopy images of Ni/Ch–2 and Ni/Ch–4 composite samples. Chitosan is known to have a thin lamellar structure with a smooth and nonporous surface [56,57]. SEM images confirmed the results presented in Table 3. The resulting composites have numerous cracks and a developed porous structure.

**Figure 7:**
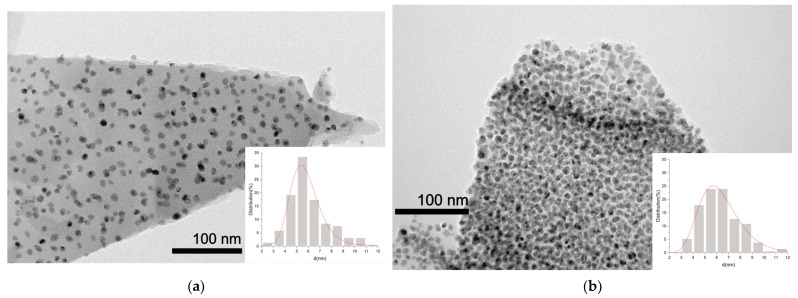
TEM images of composite materials: (**a**) Ni/Ch–2; (**b**) Ni/Ch–4.

Figure 7 shows TEM images of the Ni/Ch–2 and Ni/Ch–4 composite samples. Detailed TEM examination of the samples reveals that the resulting composites consist of uniformly distributed Ni-containing particles in a carbon matrix. For the studied samples, the particle size does not exceed 12 nm. The Ni/Ch–2 sample is characterized by a more uniform particle size distribution, as the majority are in the 4–7 nm range, compared to 4–9 nm for Ni/Ch–4. The narrower particle size distribution for the Ni/Ch–2 sample is due to the less dense organization of the Ni-containing particles in the carbon matrix.

#### 2.1.7. XPS

The qualitative and quantitative composition of the surface of the composite catalyst samples is presented in Table 4.

Table 5 presents a summary of the XPS results of the surface composition of composite materials.

The surface of the material consists of four elements: carbon, oxygen, nitrogen, and nickel. With an increase in the nickel content in the composite catalyst, a carbon matrix is formed, and for the Ni/Ch–1 (containing Ni 5 wt.% from chitosan), carbon on the surface is presented in the form of C-C (284.8 eV), C-O/sp^2^ C-N (285.6 eV), C=O/sp^3^ C-N (287.2 eV), and -COO-/CO_3_^2–^ (289.6 eV) groups [58]. The carbon matrix in the case of concentrations of 10–20 wt.% from chitosan is more graphitized—in the C 1 s spectra, there are bands from C=C (284.0–284.4 eV); it is also worth noting the π-π * shake-up (291.5–291.9 eV), which is characteristic of the formation of C=C conjugated structures [59,60]. It is worth noting separately that the surface of all composites is characterized by the presence of various oxygen-containing groups (C=O/COO-/CO_3_^2−^/C-O (285.4–289.6 eV)), which indicates significant functionalization. The nitrogen content varies and depends on the nickel content in the composite. During the preparation of the material, nitrogen is incorporated into various carbon structures—pyrrole (399.6–400.3 eV), pyridine (397.9–398.5 eV), graphite (402.7 eV), and oxidized N (pyridine–N-O) (403.0–404.4 eV) [60,61]. The nickel structure on the surface undergoes the following metamorphoses: For the Ni/Ch–1 sample, nickel on the surface is an oxidized Ni^2+^ nanoparticle (854.1, 854.9 eV), which can also be associated with nitrogen NiN_x_/C [62], which can be partially hydrated (856.1 eV) [62]. Increasing the nickel content to 10–15 wt.% from chitosan promotes the formation of a metallic phase (852.5 eV), along with the oxide phase NiN_x_/C (854.8 eV) [63]. A further increase in the nickel content (up to 20 wt.% from chitosan) leads to the formation of a more complex surface structure: along with the metallic phase (851.8 eV), the formation of a transient oxidized phase Ni^+^ (852.4 eV) is recorded [64,65]. An oxide/hydroxide layer of Ni^2+^ was also recorded (854.9 eV). The oxygen content increased upon moving from low to higher nickel concentrations. Oxygen is predominantly represented in carbon-containing functional groups (532.8 eV–533.2 eV), hydroxide groups (531.2–531.4 eV), and metal–oxide bonds, which can be attributed to the boundary of the oxide particle (529.5–530.5 eV) [66,67]. The Ni/Ch–4 sample deserves special mention. In the oxygen spectrum, an oxygen signal related to the lattice oxygen of the oxide (528.9 eV) can also be distinguished [68].

Thus, it can be concluded that the resulting material may be a hybrid Ni-N-C material, which is a system with atomically dispersed NiN_x_ active centers on an N-doped carbon matrix. By varying the nickel concentration, it is possible to actively influence the functional and chemical composition of the surface of the composite material.

### 2.2. Catalytic Activity

**Figure 8:**
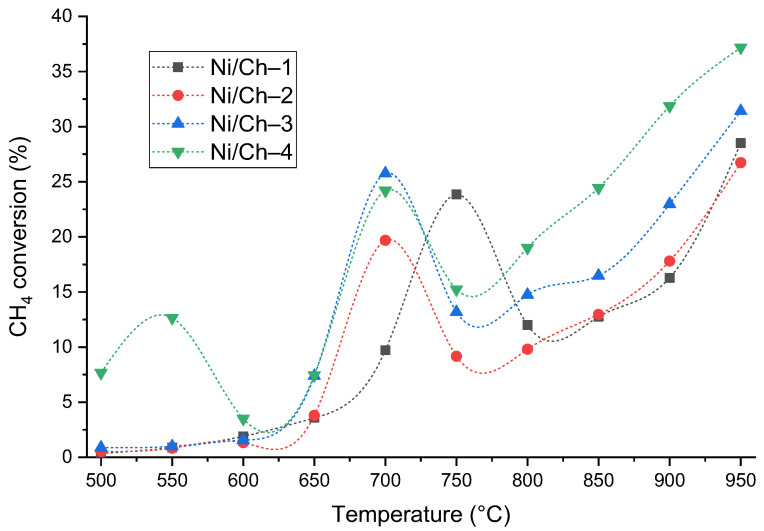
Temperature dependence of methane conversion in the methane decomposition reaction for composite material samples.

The synthesized composites are of practical interest as catalysts, which was demonstrated using the example of the thermal catalytic decomposition of methane. A key advantage of the developed materials is their ability to exhibit catalytic activity without a preliminary reduction step in a hydrogen stream, which is necessary for traditional oxide or supported Ni-containing catalysts [69,70,71]. This is made possible by the fact that the applied synthesis technique allows for the formation of the active phase of metallic nickel directly during the composite production stage. To evaluate the catalytic properties, the materials’ activity was screened in the methane decomposition reaction in the temperature range of 500–950 °C. The catalytic performance was assessed based on the methane conversion achieved after 15 min of reaction. Figure 8 shows the dependences of methane conversion on the experimental temperature for the studied samples.

An analysis of the temperature dependences of methane conversion (Figure 8) revealed general patterns and individual features of the catalytic behavior of the synthesized composites. All samples exhibited a nonmonotonic activity profile, typical of systems where activation and sintering processes compete with an increase in temperature. The initial increase in conversion (in the range of 500–650 °C) is likely associated with the activation and/or reduction of residual nickel oxide. A subsequent decrease in activity in the range of 750–800 °C is explained by the agglomeration of these particles, after which, at temperatures above 850 °C, a secondary increase in conversion is observed, probably due to the activity of the carbon product of methane decomposition in the same process. Moreover, the key differences correlate with the composition and texture of the materials. The Ni/Ch–1 sample, with the lowest Ni content, demonstrates the expected low activity in the low-temperature region. The Ni/Ch–2 and Ni/Ch–3 catalysts exhibit similar behavior, reaching maximum conversion (~24–26%) already at 700 °C, which may indirectly indicate the optimal number and dispersion of active sites. The most pronounced and complex dynamics are demonstrated by the Ni/Ch–4 sample with the highest nickel content. It is distinguished by significant initial activity at 500 °C and the presence of two conversion peaks (at 550 and 700 °C). This behavior may indicate the stepwise involvement of different forms of nickel in the process. Despite sintering at temperatures above 700 °C, this sample reaches methane conversion (37.2%) by 950 °C. However, conversion is not the only indicator of the methane decomposition process. The main process parameters at methane decomposition temperatures of 550 °C, 700 °C, and 950 °C are presented in Table 6.

In addition to methane conversion, two key parameters were calculated to evaluate the catalytic efficiency of the materials: activity (A), expressing the mass of H_2_ formed per unit mass of catalyst per hour, and hydrogen yield, characterizing the mass of H_2_ per unit mass of Ni per hour. At low temperature (550 °C), only the Ni/Ch–4 sample exhibits significant activity (0.32 g_H2_/g_cat_/h) and, most importantly, a high specific hydrogen yield at low temperature (0.68 g_H2_/g_Ni_/h). This indirectly confirms the earlier conclusion that highly dispersed and easily activated nickel-based centers are formed in the materials already at the synthesis stage, which operate effectively even under mild conditions. For the remaining samples with a lower nickel content, the activity at this stage is negligible. In the region of the methane conversion peak (700 °C), all catalysts except Ni/Ch–1 demonstrate high activity (from 0.39 to 0.62 g_H2_/g_cat_/h). The maximum yield of H_2_ is observed for the Ni/Ch–2 and Ni/Ch–3 samples (1.90 and 1.98 g_H2_/g_Ni_/h, respectively). This indicates that these composites are characterized not only by high dispersion of the active phase but also by the most efficient use of the introduced nickel. For Ni/Ch–4, despite the highest activity (0.62 g_H2_/g_cat_/h), the specific yield is significantly lower (1.30 g_H2_/g_cat_/h), which indicates a decrease in the efficiency of metal use with its excess. At high temperatures (950 °C), despite the increase in methane conversion and catalyst activity (A for Ni/Ch–4 reaches 0.95 g_H2_/g_cat_/h), the specific yield of hydrogen decreases with increasing nickel content in the composites. It can be assumed that under conditions of prolonged high-temperature exposure, carbon formed during the reaction begins to act as a catalyst [72,73]. Probably, the morphology and catalytic properties of the resulting carbon product depend on the amount of nickel in the composite. The composite with the lowest nickel content (Ni/Ch–1) demonstrates the highest specific yield under these conditions, which can be explained by the formation of an optimally structured carbon co-catalyst on its basis.

Thus, it can be concluded that the selection of the optimal composite material as a methane decomposition catalyst should be based on the process temperature used. For a low-temperature process (550 °C), the use of a composite with a high nickel content (Ni/Ch–4) is justified. For a medium-temperature process (700 °C), a material with an optimal metal balance (Ni/Ch–2 or Ni/Ch–3) is justified. And for a high-temperature process (950 °C), a system with a minimal amount of metal (Ni/Ch–1), where synergy with the forming carbon obtained from methane plays a key role.

Figure S2 (Supplementary Materials) shows the methane conversion dependence for the reduced composite catalyst samples. The Ni/Ch–3 and Ni/Ch–4 samples were chosen for the study because their TPR profiles exhibit the most pronounced hydrogen absorption peak in the 450–500 °C region, corresponding to the reduction of NiO to Ni. After preliminary reduction in a flow of H_2_, the low-temperature activity of the catalysts (up to 750 °C) decreases sharply. The conversion peaks observed for the initial composites in the 700–750 °C region completely disappear. Conversion begins to increase only above 800 °C, which is likely due to changes in the state and morphology of the active phase.

### 2.3. Characteristics of the Carbon Product

**Figure 9:**
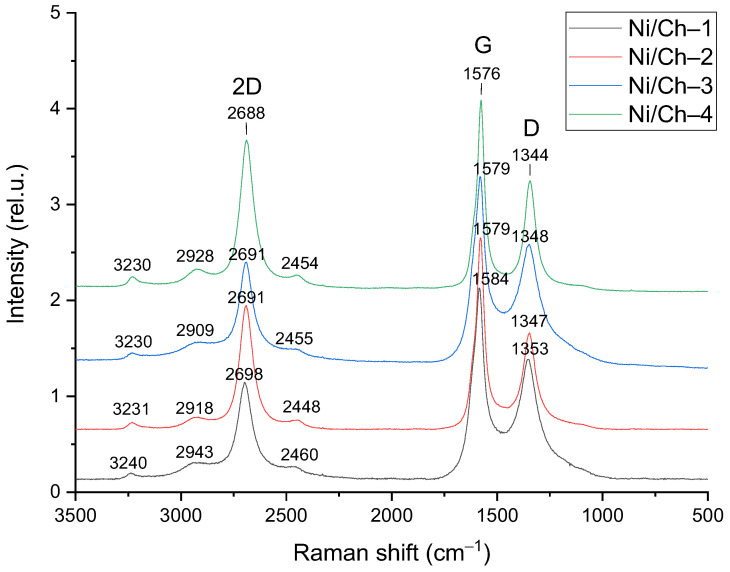
Raman spectra of the carbon product obtained on composite catalysts.

It is known that carbon of various structures can be formed during the catalytic decomposition of methane [74,75]. The Raman spectra of the obtained carbon product are shown in Figure 9. The spectra show two main peaks at about 1350 and 1580 cm^−1^, c corresponding to the D and G bands, respectively. There are also four less intense peaks in the region of 2400–3300 cm^−1^. The most pronounced second-order Raman peak is located at a frequency of ~2700 cm^−1^ and is called 2 D or G’ [76]. The obtained Raman spectra have the following distinctive features: the first-order bands are narrow, and there is also an intense second-order peak with a large number of shoulders. These distinctive features can be attributed to multi-walled carbon nanotubes (MWCNTs), which is consistent with the literature data, since in many studies, when using a nickel-containing catalyst, the carbon from methane decomposition is formed in the form of various carbon nanotubes [77,78,79].

Table 7 presents more detailed characteristics of the obtained Raman spectra. It was found that in a series of samples, an increase in the nickel content in the initial composites leads to a small but noticeable increase in the defectiveness of carbon nanotubes (an increase in the I_2 D_/I_G_ ratio from 0.56 to 0.76). The presence of an intense second-order 2 D peak indicates the formation of MWCNTs [80]. The I_2 D_/I_G_ ratio is a key parameter for indirectly assessing the number of layers in MWCNTs [81]. A higher I_2 D_/I_G_ value usually corresponds to a greater number of ordered layers. In this case, the highest I_2 D_/I_G_ value (1.34–1.43) was observed for carbon obtained on the composite with the highest Ni content (Ni/Ch–4). At the same time, the lowest I_2 D_/I_G_ ratio (0.38–0.56) was obtained for the Ni/Ch–3 composite, which may indicate the formation of nanotubes with a smaller number of layers. Interestingly, no direct linear correlation is observed between the nickel content and the I_2 D_/I_G_ ratio. This nonmonotonic behavior suggests that the number and perfection of carbon layers depend not only on the amount of active metal but also on other factors, such as the size of the nickel particles and the specific conditions of carbon deposition during growth.

## 3. Materials and Methods

### 3.1. Synthesis of Composite Materials

Composite catalysts were synthesized using matrix isolation. Chitosan was used as the carbon precursor, and nickel (II) nitrate hexahydrate (Analytical grade, Vekton, Moscow, Russia) was used for the nickel precursor.

In the first step of composite catalyst synthesis, a mixed solution of the polymer and the active metal salt was prepared. A 5 g sample of chitosan was dissolved in an aqueous acetic acid solution (0.125 M, 280 mL) with stirring and heating (45 °C), after which nickel (II) nitrate hexahydrate was added. The mixed solution was then dried to remove the solvent until a polymer film of constant weight was obtained. The nickel content in the polymer–salt mixture varied between 5 and 20% by weight of chitosan, calculated as pure nickel. The resulting film was then subjected to heat treatment in a tubular quartz furnace in an argon flow (10 L/h) for a total of 3 h: 60 °C—1 h (removal of nitric acid formed during the dissolution of nitrate salts), 100 °C—1 h (removal of residual water), and 500 °C—1 h (decomposition of nitrate and destruction of the polymer).

### 3.2. Sample Research Methods

#### 3.2.1. Elemental Analysis

The Ni content in the samples was determined by flame atomic absorption analysis. A Perkin Elmer AAnalyst 400 atomic absorption spectrometer (Perkin Elmer, Waltham, MA, USA) with a relative error of 5–8% was used. Two types of flame were used: acetylene–air (~2300 °C) and nitrous oxide–acetylene (~3000 °C). Simultaneous determination of the C, H, and N content in the samples was carried out by chromatography after combustion of the sample in a dynamic Dumas flash. A Thermo Flash 2000 elemental analyzer (Thermo Fisher Scientific, Heysham, England, UK) was used.

#### 3.2.2. Transmission and Scanning Electron Microscopy

The surface topography of the samples was assessed using a JEOL JIB-4501 scanning electron microscope (JEOL, Tokyo, Japan). A more detailed study of the sample morphology was performed using transmission electron microscopy (TEM) using a JEOL JEM 2100 electron microscope (JEOL, Tokyo, Japan).

#### 3.2.3. Raman Spectroscopy

Raman spectra were recorded using a Bruker Senterra II confocal Raman spectrometer (Bruker, Billerica, MA, USA). A laser with a wavelength of 532 nm and a power of 0.25 mW was used. For the Raman spectra, I_D_/I_G_ and I_2 D_/I_G_ were calculated based on the deconvolution results in the Fityk program.

#### 3.2.4. IR Spectroscopy

Measurement of optical reflection spectra in the IR range was carried out using a Fourier spectrometer IFS 66 v/s manufactured by Bruker (Bruker, Billerica, MA, USA).

#### 3.2.5. XRD

X-ray diffraction analysis (XRD) was performed on a Tongda TD-3700 X-ray diffractometer (Dandong Tongda Science and Technology, Dandong, China), equipped with an X-ray tube with a copper anode, a Mythen2 R 1 K linear multichannel semiconductor detector, and a Goebel mirror, an X-ray optical element for forming a parallel beam. The average crystallite size was determined from the broadening of the observed diffraction maxima using the Debye–Scherrer equation.

#### 3.2.6. The Surface Area

Specific surface area and pore size were determined using an ASAP 2020 V4.00 analyzer (Micromeritics, Norcross, GA, USA). Adsorption was carried out at −195.7 °C using N_2_ as the adsorbate. The pore size range was 0.35 to 500 nm. Specific surface area (SSA) was calculated using the Brunauer–Emmett–Teller (BET) method, and pore diameter and volume were calculated using the Barrett–Joyner–Halenda (BJH) method.

#### 3.2.7. TPR-H_2_

Temperature programmed reduction (TPR–H_2_) of catalyst samples was carried out in a 5 mm diameter quartz flow reactor. Temperature was controlled using a chromel–alumel thermocouple. The temperature programming range was from room temperature to 700 °C, under atmospheric pressure. A flow of H_2_/Ar gas containing 5 vol.% hydrogen was supplied at a rate of 50 mL/min. The hydrogen content at the reactor outlet was determined using a thermal conductivity detector on a Crystallux–4000 M chromatograph (Meta–Chrom, Yoshkar-Ola, Russia). The sample weight for all catalyst samples was 30 mg.

#### 3.2.8. XPS Spectroscopy

The study of the composite material samples’ surface was carried out by X-ray photoelectron spectroscopy on an X-ray photoelectron spectrometer (Prevac, Rogow, Poland). An X-ray tube with AlKα radiation (1486.6 eV) was used as a source of ionizing radiation. Before loading into the spectrometer, the samples were ground in an agate mortar and applied to conductive carbon tape. To neutralize the charge of the sample during the experiments, an electron–ion charge compensation system was used. All peaks were calibrated versus the C 1 s peak at 284.8 eV. The type of background was Shirley, and during deconvolution, it was assumed that the total peak was the sum of Gaussian curves.

### 3.3. Catalyst Activity Test

Catalytic tests of methane decomposition were conducted in a flow-through quartz reactor with a fixed catalyst bed. Methane (99.9 vol. %) was used as the feedstock. The reactor was set to a temperature of 500 °C (heating increments of 50 °C at 15 min intervals), after which methane was introduced (GHSV 1500 h^−1^). Each of the catalysts was tested without a preliminary activation step and under identical conditions.

The reactor outlet gas was analyzed online using a Kristallyuks-4000 M chromatograph (Meta-Chrom, Yoshkar-Ola, Russia). Separation of the gas mixture components was performed using a packed column with a Carboxen carbon adsorbent (column length 3 m; internal diameter 3 mm), with a thermal conductivity detector. The components were separated using a temperature-programmed mode (from 50 °C to 190 °C at a heating rate of 7.5 °C/min), using argon as the carrier gas (flow rate 30 mL/min). No gases other than hydrogen and methane were detected in the product gas.

The main process indicators were calculated using Equations (1)–(3).

(1) $$X_{CH4}\left(\%\right)=\frac{C_{H2}(vol.\%)/2}{C_{CH4}(vol.\%)+C_{H2}(vol.\%)/2}\cdot 100\%$$

(2) $$A\left(g_{H2}/g_{cat}/h\right)=\frac{M_{H2}(g/mol)\cdot n_{CH4}(mol/h)\cdot X_{CH4}(\%)\cdot 2}{m_{cat}(g)\cdot 100\%}$$

(3) $$Yield\left(g_{H2}/g_{Ni}/h\right)=\frac{A\left(g_{H2}/g_{cat}/h\right)}{{Content}_{Ni}(wt.\%)}\cdot 100\%$$

## 4. Conclusions

Thus, in this study, new nickel/chitosan composite materials obtained using the organic matrix method were synthesized and characterized. A key feature of the proposed approach is the formation of an active nickel metallic phase directly during composite synthesis, eliminating the need for a mandatory catalyst pre-reduction step. Comprehensive characterization of the synthesized materials confirmed the chemical interaction between nickel ions and chitosan functional groups and also allowed us to establish a relationship between nickel content and textural characteristics. It was established that nitrate ion decomposition acts as an activating agent, enabling targeted modification of the specific surface area and micropore-to-mesopore ratio in the material.

A study of the catalytic properties in the thermocatalytic decomposition of methane revealed a dependence of activity on nickel content, which is determined by the target process temperature. The selection of the optimal Ni/Ch catalyst is determined by the target process temperature. The composite with the highest nickel content (Ni/Ch–4) demonstrates the highest efficiency at low temperatures (550 °C). In the mid-temperature range (700 °C), the most efficient metal utilization is observed for the Ni/Ch–2 and Ni/Ch–3 composites, while for high-temperature conditions (950 °C), the material with the lowest nickel content (Ni/Ch–1) proves most promising.

These results demonstrate the fundamental possibility of controlling catalytic properties through targeted design of the composition and texture of the composite material. The identified synergistic effect between the metal phase and the carbon matrix, as well as the formation of an active carbon co-catalyst during the reaction, opens up prospects for further research and the development of highly efficient catalysts for methane decomposition to produce hydrogen.

## Figures and Tables

**Table 1:** Elemental analysis of composite materials.

No	Sample	Ni, wt.% from Chitosan	Content, wt.%				
			Ni	C	N	H	O
1	Ni/Ch–1	5	10.8	61.7	11.3	2.1	14.0
2	Ni/Ch–2	10	20.4	55.3	10.8	2.0	11.4
3	Ni/Ch–3	15	28.7	43.9	7.9	1.3	18.2
4	Ni/Ch–4	20	47.5	40.7	6.7	1.1	4.1

**Table 2:** Wavenumbers (cm^−1^) of main bands from FTIR spectra of the polymer (chitosan) and salt–polymer films.

No	Wavenumbers, cm^−1^					Comments
	Chitosan	Ni/Ch–1	Ni/Ch–2	Ni/Ch–3	Ni/Ch–4	
1	3366	3362	3317	3308	3311	Stretching vibrations OH and NH
2	-	3177	3180	3178	3179	
3	2914	2925	2925	2928	2927	Stretching vibrations of –CH_2_
4	2874	2851	2854	2854	2854	
5	1653	1628	1629	1617	1601	Stretching vibrations of C=O (amide I)
6	1593	1548	1543	1551	1555	Deformation vibrations of NH
7	1423	is blocked by another lane				Stretching vibrations of –CH_2_
8	1320					
9	1262	1252	1251	1256	1254	Cyclic ethers, ring stretching vibrations
10	1253	1253	1253	1253	1254	Stretching vibrations of –CH_2_
11	1153	1153	1153	1154	1155	Refers to the structure of the saccharide
12	1109	1111	1111	1113	1113	Stretching vibrations of C-O
13	1032	1032	1025	1025	1025	
14	947	945	945	946	947	Cyclic ethers, ring stretching vibrations
15	896	896	894	896	894	
16	-	827	827	827	827	Nitro compounds R-NO_2_

**Table 3:** Textural characteristics of composite materials.

No	Sample	S_sp_, m^2^/g	S_meso_, m^2^/g	S_micro_, m^2^/g	S_ext_, m^2^/g	V_pore_, mL/g	V_meso_, mL/g	V_micro_, mL/g	D, nm
1	Ni/Ch–1	26	1	24	1	0.03	0.00	0.03	4.0
2	Ni/Ch–2	257	9	244	4	0.14	0.03	0.11	2.2
3	Ni/Ch–3	269	44	206	19	0.25	0.15	0.10	3.7
4	Ni/Ch–4	224	63	132	29	0.30	0.24	0.06	5.3

**Table 4:** Qualitative and quantitative surface composition of composite materials.

No	Sample	Content	Element			
			Ni	C	N	O
1	Ni/Ch–1	at.%	1.2	82.2	9.1	7.5
2	Ni/Ch–2	at.%	1.8	78.9	10.0	9.3
3	Ni/Ch–3	at.%	3.2	79.5	8.4	8.9
4	Ni/Ch–4	at.%	4.8	78.2	6.2	10.8

**Table 5:** Summary of XPS results of the surface composition of composite materials.

No	Sample	Element	Binding Energy, eV	FWHM, eV	%	Compound Type
1	Ni/Ch–1	C 1 s	285.6	2.16	39.6	C-O/sp^2^ C-N
			284.8	1.54	40.0	C-C
			289.6	4.23	7.7	-COO-/CO_3_^2−^
			287.2	3.32	12.7	C=O/sp^3^ C-N
		N 1 s	399.8	2.14	41.4	Pyrrole nitrogen
			397.9	1.90	52.9	Pyridinic nitrogen
			402.7	3.05	5.7	Tetracoordinate nitrogen
		Ni 2 p_3/2_	861.0	6.10	28.3	Satellite
			854.9	1.79	14.3	Ni^2+^
			854.1	3.70	49.8	Ni^2+^
			856.1	1.97	7.7	Ni(OH)_2_
		O 1 s	532.8	3.369	66.1	C=O/COOH/C-O
			531.2	2.024	24.7	OH
			530.4	2.60	9.2	Me-O
2	Ni/Ch–2	C 1 s	291.5	2.53	2.2	π-π * shake-up
			284.0	1.49	15.8	C=C
			288.7	3.27	10.3	C=O/C(O)O-
			284.5	1.26	22.4	C-C
			286.8	1.89	10.9	C-O-C/C-OH
			285.4	1.76	38.5	C-O/sp^2^ C-N
		N 1 s	400.3	2.10	39.4	Pyrrole nitrogen
			398.5	1.81	49.5	Pyridinic nitrogen
			403.0	5.47	11.1	Oxidized N (pyridine-N-O)
		Ni 2 p_3/2_	854.8	2.98	52.0	Ni^2+^
			852.5	1.51	17.2	Ni met
			859.7	7.53	30.8	Satellite
		O 1 s	531.4	1.64	22.7	OH/C-O
			532.9	3.11	63.3	C=O/COOH/C-O
			530.5	2.03	14.0	Me-O
3	Ni/Ch–3	C 1 s	284.3	1.50	35.6	C=C
			285.0	2.02	35.0	C-C
			291.8	3.97	3.0	π-π * shake-up
			287.4	3.59	11.5	C=O/sp^3^ C-N
			286.1	2.07	9.0	C-O-C/C-OH/sp2 C-N
			289.1	3.87	5.9	-COO-/CO_3_^2−^
		N 1 s	399.6	3.13	68.9	Pyrrole nitrogen
			398.2	1.40	19.5	Pyridinic nitrogen
			404.3	6.27	11.6	Oxidized N (pyridine-N-O)
		Ni 2 p_3/2_	853.8	3.96	54.1	Ni^2+^
			852.5	1.32	28.3	Ni met
			858.7	5.07	17.6	Satellite
		O 1 s	531.2	2.41	67.3	OH/C-O
			533.2	2.47	29.3	C=O/COOH/C-O
			529.5	0.99	3.4	Me-O
4	Ni/Ch–4	C 1 s	285.7	1.90	45.6	C-O-C/C-OH/sp^2^ C-N
			284.8	1.23	16.2	C-C
			289.6	2.02	6.5	-COO-/CO_3_^2−^
			287.3	2.09	11.7	C=O/sp^3^ C-N
			291.9	1.10	0.8	π-π * shake-up
			284.4	1.50	19.3	C=C
		N 1 s	399.7	2.72	57.9	Pyrrole nitrogen
			398.0	1.51	30.6	Pyridinic nitrogen
			404.4	4.88	11.6	Oxidized N (pyridine-N-O)
		Ni 2 p_3/2_	851.8	1.17	18.1	Ni met
			852.4	2.95	48.9	Ni^+^
			857.9	5.29	22.5	Satellite
			854.9	2.04	10.5	NiO/Ni(Ni(OH)_2_)
		O 1 s	530.3	1.62	24.1	Me-O
			532.9	2.69	42.2	C=O/COOH/C-O
			531.4	1.83	23.5	-OH
			528.9	1.41	10.3	Me-O

**Table 6:** Main indicators of catalytic decomposition of methane in the presence of composite catalysts based on chitosan.

No	Temperature, °C	Sample	X_CH4_, %	A, g_H2_/g_cat_/h	Yield, g_H2_/g_Ni_/h
1	550	Ni/Ch–1	0.8	0.01	0.09
		Ni/Ch–2	0.8	0.02	0.08
		Ni/Ch–3	1.0	0.02	0.08
		Ni/Ch–4	12.7	0.32	0.68
2	700	Ni/Ch–1	9.7	0.11	0.99
		Ni/Ch–2	19.7	0.39	1.90
		Ni/Ch–3	25.8	0.57	1.98
		Ni/Ch–4	24.2	0.62	1.30
3	950	Ni/Ch–1	9.7	0.32	2.91
		Ni/Ch–2	26.7	0.53	2.58
		Ni/Ch–3	31.7	0.69	2.41
		Ni/Ch–4	37.2	0.95	2.00

**Table 7:** Characteristics of Raman spectra of the carbon product of methane decomposition on composite catalysts.

No	Sample	I_D_/I_G_	I_2 D_/I_G_
1	Ni/Ch–1	0.56–0.57	0.60–0.69
2	Ni/Ch–2	0.57–0.63	0.87–0.91
3	Ni/Ch–3	0.53–0.68	0.38–0.56
4	Ni/Ch–4	0.71–0.76	1.34–1.43

## Data Availability

The raw data supporting the conclusions of this article will be made available by the authors upon request.

## Supplementary Materials

The following supporting information can be downloaded at https://www.mdpi.com/article/10.3390/ijms27031255/s1.

## Abbreviations

The following abbreviations are used in this manuscript:

CDM	Catalytic decomposition of methane
SEM	Scanning electron microscopy
TEM	Transmission electron microscopy
XRD	X-ray diffraction
FTIR	Fourier transform infrared spectroscopy
TPR–H_2_	Temperature programmed reduction
SSA	Specific surface area
BET	Brunauer–Emmett–Teller
BJH	Barrett–Joyner–Halenda
Ni/Ch	Composites synthesized by the matrix isolation method
GHSV	Gas hourly space velocity, h^−1^
MWCNTs	Multi-walled carbon nanotubes
S_sp_	Surface area, m^2^/g
S_meso_	Mesopore surface area, m^2^/g
S_micro_	Micropore surface area, m^2^/g
S_ext_	External surface area, m^2^/g
V_pore_	Total pore volume, mL/g
V_meso_	Mesopore volume, mL/g
V_micro_	Micropore volume, mL/g
D	Pore size, nm
X_CH4_	Methane conversion, %
A	Activity, g_H2_/g_Cat_/h
$C_{H2}$	Hydrogen content, vol. %
$C_{CH4}$	Methane content, vol. %
