# Supplementary material for: Catalytic Design of Matrix-Isolated Ni/Chitosan Composites for Methane Decomposition
Source: Int J Mol Sci. 2026 Jan 27;27(3):1255. doi: 10.3390/ijms27031255 (PMC12898850; doi:10.3390/ijms27031255)
Supplement: Supplementary file 1 [file ijms-27-01255-s001.zip › ijms-4084462-supplementary.pdf]

Supplementary Materials for the article

Catalytic Design of Matrix-Isolated Ni/Chitosan Composites for Methane Decomposition

A. Sotnikova*, M. Ivantsov, V. Vasileva, M. Kulikova

Topchiev Institute of Petrochemical Synthesis, Russian Academy of Sciences, Leninsky Prospekt, Bld. 29, 119991, Moscow, Russia;
E-mail: sotnikova.anast@ips.ac.ru

CONTENTS

1. FTIR of salt-polymer films 3
2. Catalytic activity of reduced composites 4

1. FTIR of salt-polymer films

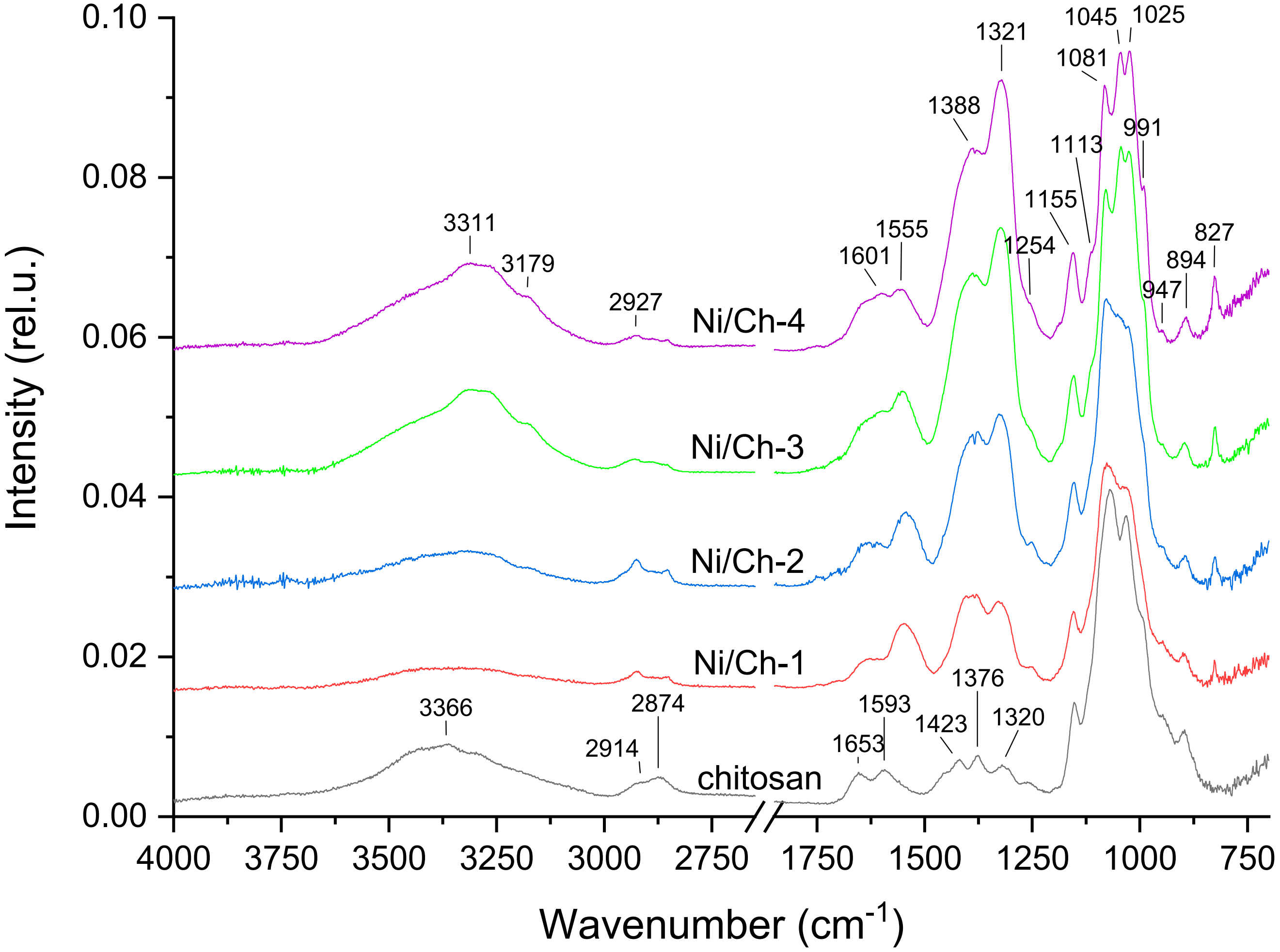

Figure S1. FTIR of chitosan and salt-polymer films

2. Catalytic activity of reduced composites

Catalytic tests of methane decomposition were conducted in a flow-through quartz reactor with a fixed catalyst bed. The composites were reduced with hydrogen (GHSV 1500 h^{-1}) at 500 °C for 1 h. Methane (99.9 vol. %) was used as the feedstock. The reactor was set to a temperature of 500 °C (heating increments of 50 °C at 15 min intervals), after which methane was introduced (GHSV 1500 h^{-1}). Each of the catalysts tested was tested without a preliminary activation step and under identical conditions.

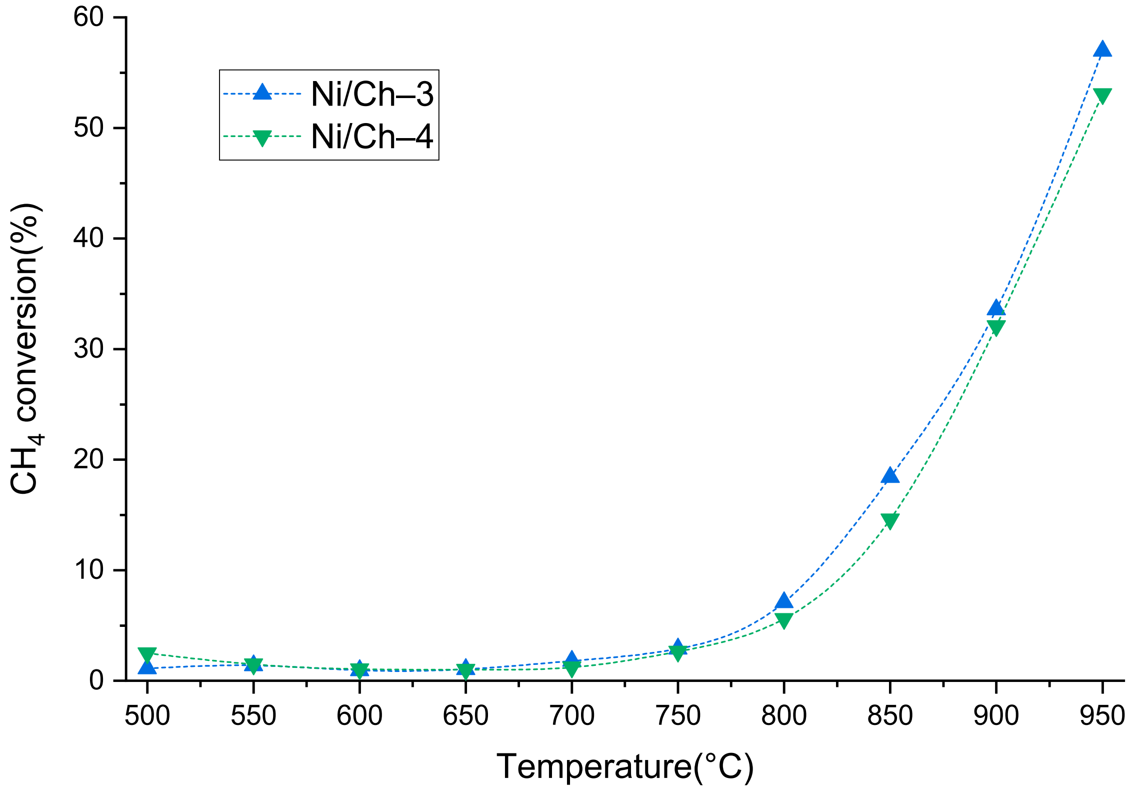

Figure S2. Temperature dependence of methane conversion in the methane decomposition reaction for reduced composite material samples.
